# Neuronal activity-dependent gene expression is stimulus-specific and changes with neuronal maturation

**DOI:** 10.3389/fnmol.2025.1609772

**Published:** 2025-10-09

**Authors:** Jeronimo Lukin, Maximiliano S. Beckel, Olivia Pedroncini, Sebastian A. Giusti, Giuliana C. Di Mauro, Ivana Marcela Linenberg, Ines Lucia Patop, Ariel Chernomoretz, Antonia Marin-Burgin, Sebastian Kadener, Damian Refojo

**Affiliations:** ^1^Instituto de Investigación en Biomedicina de Buenos Aires (IBioBA) - CONICET - Partner Institute of the Max Planck Society, Buenos Aires, Argentina; ^2^Fundación Instituto Leloir, Buenos Aires, Argentina; ^3^IIBBA CONICET, Buenos Aires, Argentina; ^4^Department of Biology, Brandeis University, Waltham, MA, United States; ^5^Departamento de Física, FCEN, Universidad de Buenos Aires and INFINA (UBA-CONICET), Buenos Aires, Argentina; ^6^Molecular Neurobiology, Max Planck Institute of Psychiatry, Munich, Germany

**Keywords:** neuronal development, activity-regulated gene expression, immediate-early genes, comparative transcriptomics, KCl, Bicuculline, TTX withdrawal

## Abstract

**Introduction:**

Neuronal activity-dependent gene expression is fundamental to a wide variety of brain functions. The field of neuronal activity-induced gene expression has advanced greatly due to studies performed in early neuronal cultures (7 to 10 DIV) and stimulated with different activation protocols. However, the effect of the developmental stage as well as the influence of specific protocol stimuli like potassium chloride (KCl)-induced depolarization, bicuculline (Bic)-mediated synaptic activation and TTX-withdrawal (TTXw) on activity-induced transcription has not been systematically studied.

**Methods:**

To analyze the influence of neuronal maturation on activity-induced transcription, we used neuronal primary cultures to compare electrophysiological and transcriptional responses at 7 days *in vitro* (DIV) and 21 DIV upon KCl and Bic stimulation. Also, mature neurons in culture were subjected to treatments with KCl, Bic and TTXw and the transcriptional changes were assessed by RNA-Seq and *post-hoc* bioinformatic analysis.

**Results:**

Our results demonstrate that the developmental stage of neurons profoundly influences neuronal firing and gene expression. The response to KCl and Bicuculline was dramatically different, even though these compound-based activation protocols have been widely used and considered as methods that produce equivalent effects. Therefore, we next asked how 21DIV neurons, more advanced in their development, react to different stimuli and observed that KCl, Bic and TTXw, which trigger different firing patterns, induce specific transcriptional profiles with unique temporal dynamics and activating a variety of gene groups.

**Conclusion:**

These findings hold both technical and conceptual significance. Technically, they underscore the importance of accounting for neuronal maturation and activation protocols when studying gene expression. Conceptually, they demonstrate that neuronal development and drug-induced firing patterns generate distinct expression profiles, which could be crucial for a deeper understanding of transcription-dependent plasticity mechanisms.

## Introduction

Activity-dependent gene expression is a molecular mechanism in neurons crucial for multiple cognitive, sensory-motor, developmental, and emotional adaptive processes (Benito et al., 2011; Yap and Greenberg, 2018). Immediate early genes (IEGs) are rapidly induced upon neuronal stimulation in a protein synthesis-independent manner (Fowler et al., 2011; Greenberg et al., 1985; Morgan et al., 1987). Numerous studies have demonstrated that various external stimuli activate the expression of IEGs, including growth factors (Cochran et al., 1983; Cole et al., 1989), neurotrophins (Ghosh and Greenberg, 1995; Martinowich et al., 2003), glutamate (Bading et al., 1993), NMDA (Pappas et al., 2012), electrical stimulations (Lee et al., 2017), potassium chloride (KCl)-induced depolarization (Carullo et al., 2020; Kim et al., 2010; Malik et al., 2014; Tyssowski et al., 2018), Bicuculline (Bic) (Bas-Orth et al., 2017; Benito et al., 2011; Rienecker et al., 2022; Tyssowski et al., 2018; Zhang et al., 2007), and Tetrodotoxin withdrawal (TTXw) (Rao et al., 2006; Saha et al., 2011). From these studies, specific IEGs, such as Fos, Arc, Npas4, Homer1, Igf1, and Bdnf, have been further characterized for their roles in neuronal function and plasticity (Barth et al., 2004; Bateup et al., 2013; Diering et al., 2017; Hu et al., 2010; Jakkamsetti et al., 2013; Joo et al., 2016; Mardinly et al., 2016; Rial Verde et al., 2006; Shepherd and Bear, 2011; Spiegel et al., 2014; Sun and Lin, 2016). Importantly, not all IEGs respond to every activation stimulus, and the mechanisms underlying these differences are not yet fully understood.

The impact of electrical activity on neuronal development and connectivity is well documented, both in cell culture and *in vivo* paradigms (Andreae and Burrone, 2014; Fields and Nelson, 1992; Goodman and Shatz, 1993; Martens et al., 2016; Shatz, 1990; Spitzer, 2006; Valor et al., 2007). However, there is a significant knowledge gap in systematic research regarding how activity-dependent gene expression programs vary at different stages of maturation. Much of our knowledge about IEG mechanisms originates from studies conducted on primary neuron cultures stimulated at 7–10 days *in vitro* (DIV). However, at this developmental stage, neurons are not fully mature, raising the question about the influence of neuronal development on activity-induced gene expression.

The temporality of neuronal activation is another critical factor for shaping activity-dependent gene expression programs. While increased frequencies of electrical stimulation correlate with increased expression levels of c-fos (Sheng et al., 1993), the duration of neuronal activation also plays a significant role in shaping gene expression (Lee et al., 2017; Tyssowski et al., 2018). Moreover, specific bursting patterns of neuronal activation exert differential effects over gene expression profiles, even when neurons receive the same number of electric stimulations (Iacobas et al., 2019; Lee et al., 2017; Sheng et al., 1993). However, these studies utilized prolonged activation protocols (up to 5 h), potentially confounding interpretation due to homeostatic phenomena. Additionally, these studies were performed in dorsal root ganglion (DRG) neurons, which remain silent in culture and lack dendrites and synaptic contacts. Consequently, the impact of acute activation patterns on gene expression in cultured cortical neurons needs to be further elucidated.

Here, we investigate the influence of neuronal development on the transcriptional response to activity by conducting a comparative analysis of the activity-induced gene expression between 7DIV and 21DIV neurons in culture stimulated by synaptic activity (Bic) and massive depolarization (KCl). Additionally, to address how different activity patterns influence gene transcription in neurons, we performed a comparative analysis of global gene expression in neurons acutely activated with KCl, Bic, and TTX withdrawal. Overall, our findings suggest that the transcriptional response to activity-driven stimulation is strongly influenced by the progression of neuronal development and that different modalities of neuronal activity elicit specific transcriptional programs with unique and distinct temporal dynamics.

## Methods

### Mouse primary neuronal cultures

Neurons were dissected from embryonic day 16.5 (E16.5) CD1 embryos of mixed sex. Culture preparation was performed as previously described, obtaining neuronal enriched cultures with minimal glial contribution (Giusti et al., 2024). Briefly, cortex and hippocampus from CD1 mouse embryos were dissected to focus on a broad transcriptional response to neuronal activity rather than region-specific effects. Neuronal suspension was prepared through Trypsin digestion and mechanical disruption of the tissue. Neurons were plated in 24 multi-well plates at a density of 80 cells/mm2 (150.000 cells per well) and maintained in neuronal maintenance medium containing Neurobasal-A media (ThermoFisher) with 2% B27 and 0.5 mMGlutaMAX-I (ThermoFisher) at 37 °C and 5% CO2. CD1 mice were provided by our Specific Pathogen Free Animal Facility. All procedures were done in accordance with local regulations and the NRC Guide for the Care and Use of Laboratory Animals, followed at IBioBA-CONICET and approved by the local Institutional Animal Care and Use Committee (Protocol number 2020-02-NE) and were following the general guidelines of the National Institute of Health (NIH, USA).

### Stimulation protocols

Neurons were incubated in 1mL of neuronal maintenance medium and used either at 19–23 DIV (“21DIV”) or at 7DIV when indicated. Stimulations were performed in the same medium, by adding drugs at the final concentrations specified. No previous silencing was applied in any of the stimulation protocols. Massive membrane depolarization was achieved by applying 55 mM extracellular potassium chloride (KCl). We triggered neuronal activity by treating neurons with 50 μM Bicuculline (Sigma) to induce synaptic stimulation. As indicated in the 7 DIV−21 DIV comparison, we also added 2.5 mM 4-Aminopyridine (Sigma) to the 50 μM Bicuculline treatment. To prepare the stimulation solutions, 50 μL of conditioned medium was removed from each well and replaced with 50 μL of a 20X concentrated KCl or Bicuculline solution respectively to achieve the final treatment concentration. Basal wells were handled in the same way, except that no drug was added.

Synaptic rebound was induced by performing TTX withdrawal (Rutherford et al., 1997; Saha et al., 2011; Turrigiano et al., 1998). Cultures were treated with 1 μM TTX (Tocris) for 48 hs, and the TTX was then washed out through seven exchanges of 1 ml of medium with fresh control medium. Control neurons were washed identically and processed in parallel. Results are shown in comparison to TTX control situation (silenced neurons TTX, 48 hs). Each stimulation was sustained until the indicated time.

### RNA sequencing and analysis

RNA from primary neuron cultures RNA was extracted using the RNeasy mini kit (QIAGEN) with in-column DNase treatment (QIAGEN) according to the manufacturer's instructions.

15–25 ng of RNA were used as input for preparing 3′ RNA sequencing libraries following CelSeq2 protocol (Hashimshony et al., 2016), changing the UMI to six bases. Sequencing was performed on Illumina NextSeq 500 system. Raw reads were aligned to Mus musculus genome (version mm10) using STAR (Dobin et al., 2013). Reads were quantified using End Sequence Analysis Toolkit (Derr et al., 2016) for 3′ RNA libraries. Three experimental replicates were made in most of the conditions (with an *n* = 2 and *n* = 4 in some specific time-points). Differential gene expression analysis was performed with DESeq2 R package. Differential gene expression analysis was done with edgeR (Robinson et al., 2010). We excluded from the analysis genes that do not reach 5 Counts per million (CPMs) mapped reads in at least two samples. Reads were normalized by the trimmed mean method (TTM) for each time point before differential expression analysis. Significant differential expression used a cutoff of FDR < 0.05 and fold-change of at least ±1.5. Genes were grouped and ordered according to similar behaviors and dynamics through the Ward. D2 algorithm of the R package called Hierarchical Clustering when presenting gene expression levels in heatmaps.

### Data availability

The bulk RNA-seq data generated in this study are publicly available at Gene Expression Omnibus (GEO) with accession number GSE277512. https://www.ncbi.nlm.nih.gov/geo/query/acc.cgi?acc=GSE277512.

### Gene ontology enrichment analysis and gene clustering

Enrichment analyses were performed using the EnrichmentBrowser package (Geistlinger et al., 2016). For the GO analysis, categories with a number of genes between 20 and 200 were considered. Those categories with FDR-adjusted *p-value* < 0.05 were considered significant.

Clustering of gene time profiles was made from a distance matrix calculated as one minus the correlation of gene expression values. Only genes that were significant in at least one time point were considered. We considered the dynamic-tree cut method (Langfelder et al., 2008) to infer clusters from the obtained hierarchical dendrogram. In addition, a merging step was made to join the clusters whose mean profiles had a distance less than 0.3.

### Electrophysiological recordings

For electrophysiological recordings neuronal cultures were performed as described above, but cells were seeded on coverslips. Each coverslip was transferred to a chamber containing Artificial Cerebrospinal Fluid solution (ACSF) (in mM): 125 NaCl, 2.5 KCl, 2.3 NaH_2_PO_4_, 25 NaHCO_3_, 2 CaCl_2_, 1.3 MgCl_2_, 1.3 Na^+^ ascorbate, 3.1 Na^+^ pyruvate, and 10 dextrose (315 mOsm). Continuous bubbling with 95% O_2_/5% CO_2_ was administrated to the bath. Recordings were made in neurons of 7DIV and 21DIV. Whole cell current clamp recordings were performed using microelectrodes (4–6 MΩ) with an internal solution of potassium gluconate (in mM): 120 potassium gluconate, 4 MgCl2, 10 HEPES buffer, 0.1 EGTA, 5NaCl, 20KCl, 4ATP-tris, 0.3 GTP-tris, and 10 phosphocreatine (pH = 7.3; 290 mOsm). Recordings were obtained using Multiclamp 700B amplifiers (Molecular Devices), digitized and acquired at 20 kHz on a desktop computer using pClamp10 software (Molecular Devices). Spontaneous activity was recorded in current clamp mode before and during the infusion of the drugs to the bath. For KCl treatment, the resulting firing rate was calculated during the short period of depolarization compared to the first 30 s of the recording in basal conditions. Non-stimulated recordings were performed for 60 s. For Bic treatment, 45% of evaluated neurons were responsive to the treatment. In those neurons, we compared a 40 s window after the neurons are exposed to Bic with the first 30 s of the recordings in basal conditions. KCl and Bic were directly infused by pipetting a small volume into the bath reaching the final desired concentration in each case. When a drug was infused into the bath, only one neuron per coverslip was recorded to ensure that the neurons had not been previously stimulated. For TTXw recordings, coverslips were transferred to a TTX containing chamber. Spontaneous activity was recorded in current clamp mode before and after perfusing the neurons with ACSF to washed out TTX from the bath. Membrane capacitance and input resistance were obtained from current traces evoked by a hyperpolarizing step of 10 mV. Series resistance was typically 10 to 20 MΩ, and neurons were discarded if it exceeded 40 MΩ. To measure miniature excitatory postsynaptic currents (mEPSCs), recordings were performed in voltage clamp at −70 mV in the presence of 0.5 μM TTX in the bath.

### Western blots

Cells and tissue were lysed in RIPA buffer containing protease inhibitors (Roche). Protein samples were separated by 8%−10% SDS-PAGE and transferred to 0.2-μm PVDF membranes (Millipore). Chemiluminescence signal was acquired in a ChemiDoc station (BioRad) and analyzed using Image J. For ERK and phosphoERK detection the following primary antibodies were used at a 1:1000 dilution: rabbit Phospho-p44/42 MAPK -Erk1/2- (Cell Signaling Technology #9101) and rabbit p44/42 MAPK -Erk1/2- (Cell Signaling Technology #4695). Secondary antibody anti-rabbit IgG, HRP-linked Antibody (Cell Signaling Technology #7074) was used at a 1:4000 dilution.

### Immunofluorescence staining

Immunofluorescence staining were performed as previously described (Refojo et al., 2011). In brief, neuronal cultures were fixed with pre-warmed 4% paraformaldehyde containing 5% sucrose for 20 min at room temperature and then washed with phosphate-buffered saline (PBS). Samples were permeabilized and blocked in 5% BSA (Sigma Aldrich), incubated with primary antibodies (overnight at 4 °C), followed by Alexa dye-conjugated secondary antibodies (Invitrogen). Samples were mounted in VectaShield medium (Vector Laboratories). Primary antibodies used were used at a 1:100 dilution: anti-Synaptophysin rabbit policlonal (Abcam, ab 14692) and anti MAP2 chicken (Abcam, ab 5392). Secondary antibodies were used 1:2000: Goat anti-rabbit IgG (H+L) Alexa Fluor 594 (Invitrogen, A-11037) and Goat anti-Chicken IgY (H+L), Alexa Fluor^®^ 647 (Invitrogen, A-21449).

## Results

### The maturational stage of neurons influences neuronal firing and dictates the course of activity-driven transcription

Several studies have shown how basal transcriptional profiles change as neurons develop and their synaptic contacts increase and mature (Martens et al., 2016; Valor et al., 2007). However, how the degree of neuronal differentiation impacts the transcriptional programs induced by neuronal activity has not been systematically evaluated so far. To address this question, we performed an RNA-seq analysis after stimulating either 7DIV or 21DIV neurons with two different activation modalities: synaptic activity by treating cells with Bic and membrane depolarization with KCl at different time points.

**Figure 1:**
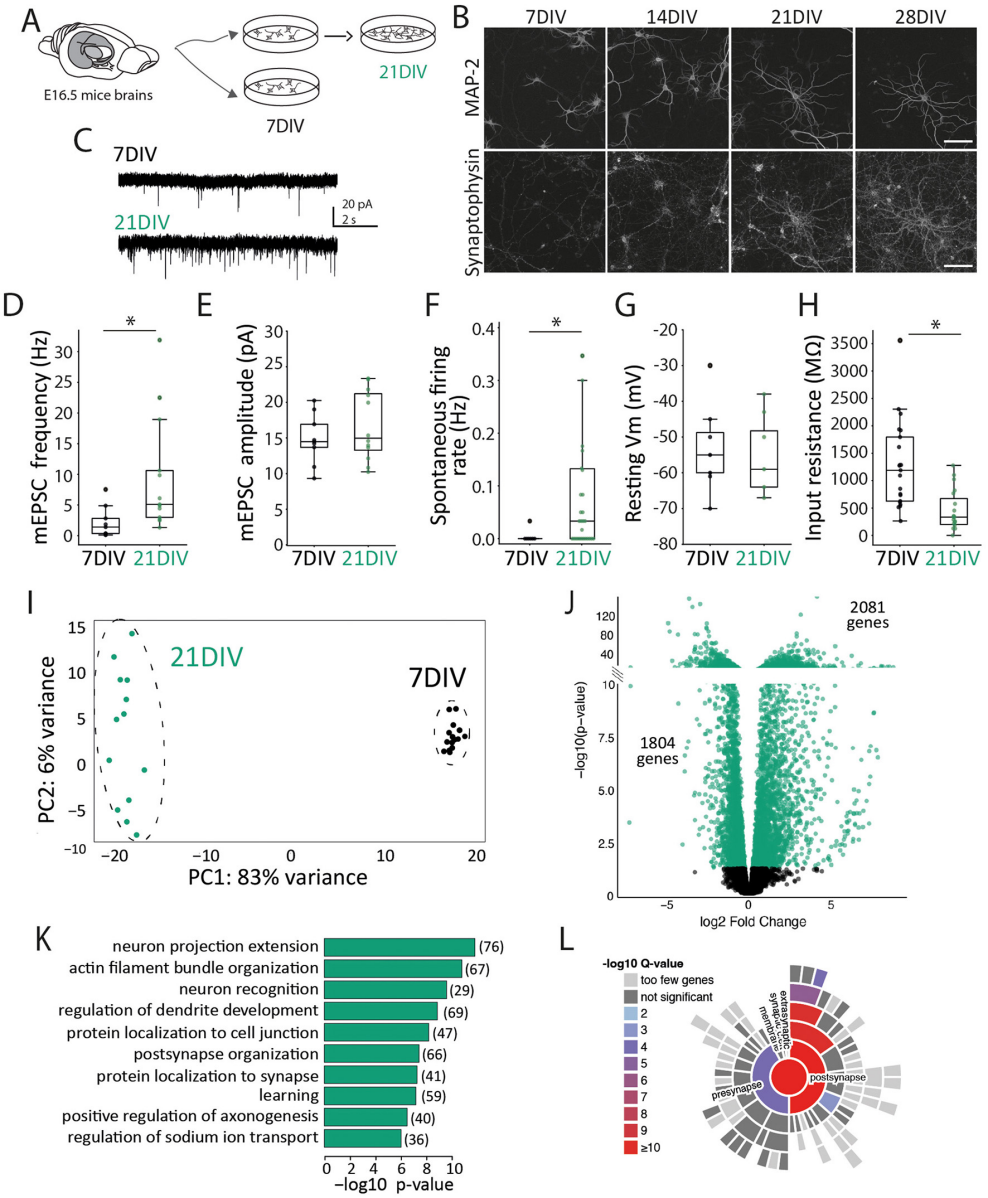
Developmental stage influences different neuronal molecular characteristics. **(A)** Experimental design: primary cortical neurons from E16.5 mouse brains were cultured for 7DIV (developing) and 21DIV (mature). **(B)** Immunocytochemistry of MAP2 and Synaptophysin at different developmental stages. Scale bar = 100 um. **(C)** Representative current-clamp recordings at 7DIV during KCl administration. Scale: *t* = 50 s. **(D)** Miniature excitatory postsynaptic currents (mEPSCs) frequency measured in 7DIV neurons (*n* = 9) compared to 21DIV neurons (*n* = 13). Each dot represents a single neuron measurement. Independent student's test: t-statistic = −2.293, *p-value* = 0.0329. **(E)** Miniature excitatory postsynaptic currents (mEPSCs) amplitude measured in 7DIV neurons (*n* = 9) compared to 21DIV neurons (*n* = 12). Each dot represents a single neuron measurement. Independent student's test: t-statistic = −0.956, *p-value* = 0.3509. **(F)** Spontaneous firing rate measured by patch clamp recordings in 7DIV neurons (*n* = 7) compared to 21DIV neurons (*n* = 30). Each dot represents a single neuron measurement. Independent Welch's *t*-test: t-statistic = −2.548, *p-value* = 0.0111. **(G)** Resting membrane potential measured by patch clamp recordings in 7DIV neurons (*n* = 12) compared to 21DIV neurons (*n* = 8). Each dot represents a single neuron. Independent student's test: t-statistic = −0.956134, *p-value* = 0.3509. **(H)** Input resistance measured by patch clamp recordings in 7DIV neurons (*n* = 21) compared to 21DIV neurons (*n* = 19). Each dot represents a single neuron measurement per well. Independent student's test: t-statistic = 4.134, *p-value* = 0.0002. **(I)** Principal Component Analysis (PCA) of gene expression data from stimulated or unstimulated samples at 7DIV and 21DIV. 7DIV *n* = 14; and 21DIV *n* = 13. **(J)** Vulcano plot showing differentially Expressed Genes (DEG) 7DIV and 21DIV in basal conditions. The plot is divided, and the scale is adapted to accommodate the broad range of *p-values*. **(K)** Gene ontology enrichment analysis. Enrichment in biological processes related to neuronal development and synapse function. The number of DEGs in each biological process is reported in brackets. **(L)** SynGO enrichment plot: gene expression differences between 7DIV and 21DIV show an enrichment in genes related to synaptic cellular components. ^*^means that the differences between the 2 groups are significant with a *p* value < 0.05.

First, by evaluating 7DIV and 21DIV unstimulated neurons (Figure 1A) we observed that neurons exhibit a more complex morphology and a higher synaptophysin expression, validating the expected increase of synaptic contacts as maturation progresses (Figure 1B). In this line, neuronal maturation was also evident by a significant increase in the frequency of mEPSCs at 21DIV (Figures 1C–E). Accordingly, whole-cell patch-clamp recordings demonstrated that neurons at 7DIV displayed a low spontaneous firing rate, which significantly increased at 21DIV (Figure 1F), evidencing higher levels of network connectivity and basal activity in 21DIV cultures. Additionally, electrophysiological recordings revealed comparable resting membrane potentials at both stages (Figure 1G), but a significantly lower input resistance at 21DIV (Figure 1H), indicative of increased membrane conductance that occurs with cellular maturation.

When performing a comparison of gene expression levels of stimulated and unstimulated neurons by RNA sequencing, the PCA analysis showed an apparent clustering of the samples belonging to different developmental stages (Figure 1I). We observed 3,885 differentially expressed genes (DEG) between 21DIV and 7DIV neuronal basal gene expression (Supplementary Table S1) with 2,081 genes significantly up-regulated and 1,804 genes down-regulated in 21DIV compared to 7DIV (Figure 1J). Gene Ontology (GO) enrichment analysis on 21DIV vs. 7DIV DEG revealed that the significantly enriched terms were related to the neuronal morphological maturation and synaptic organization and function (Figure 1K). 572 of these 3,885 DEG were mapped to unique SynGO annotated genes presenting a significant enrichment in synaptic cellular components (Figure 1L). To remark, we observed an increase in mRNA levels of glutamate receptor genes (Grin2c, Grin3, Grm1, Grin2a, Grid2, Grin3a, Grid3) as well as GABA-A receptor genes (Gad1, Gad2, Gabra1, Gabra3) (Supplementary Table S1).

**Figure 2:**
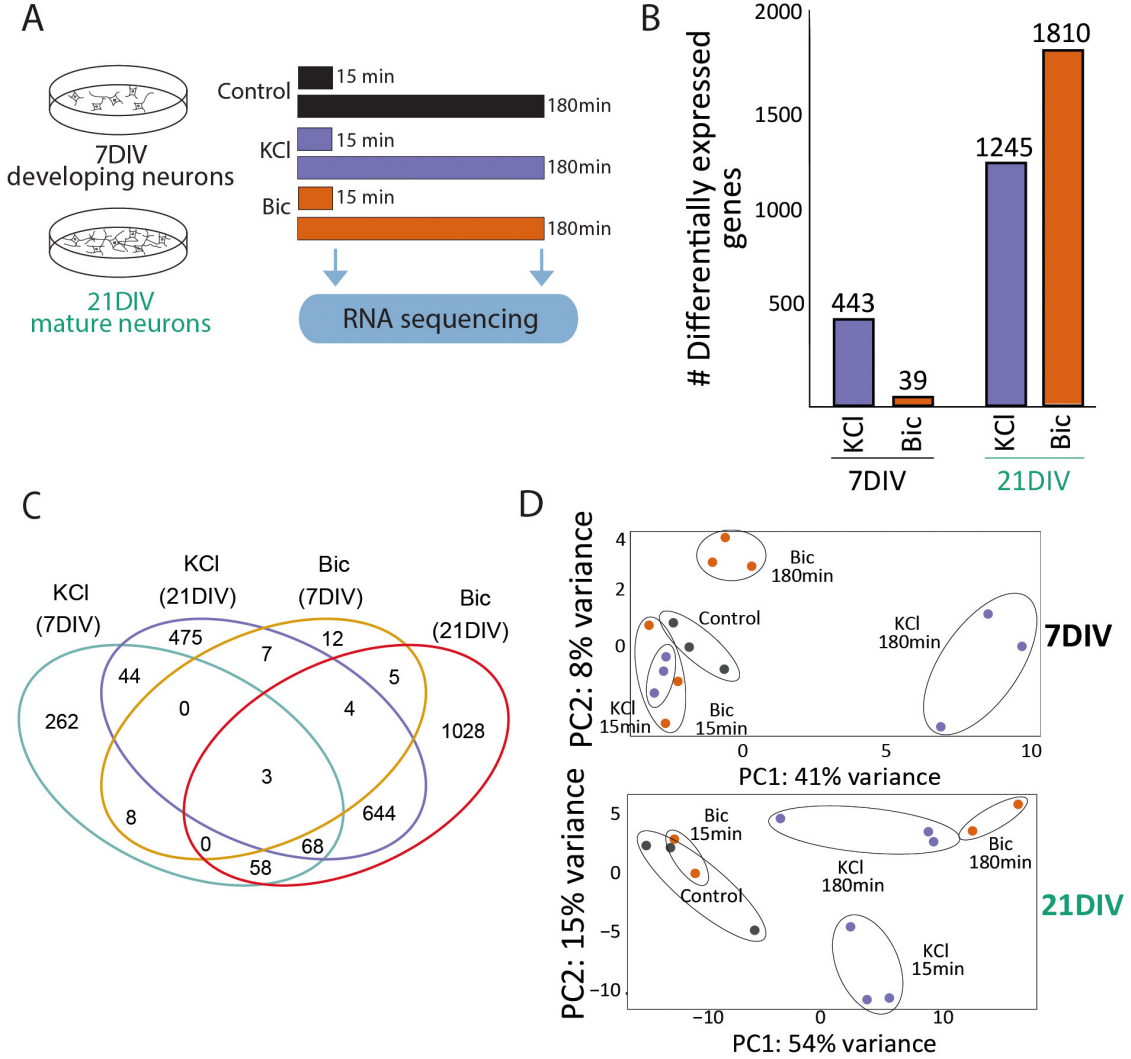
Activity-driven gene expression responses differences in 7DIV and 21DIV neurons. **(A)** Experimental design: 21DIV neurons were stimulated with KCl or Bic for 15 min or 180 min, and RNA was extracted and sequenced to assess differential gene expression. **(B)** Differentially expressed genes (DEG): number upon KCl of Bic stimulation at 7DIV and 21DIV. **(C)** Venn diagram showing the number of overlapping DEG among experimental situations. **(D)** PCA of gene expression data from 7DIV (top) or 21DIV (bottom) stimulated neurons shows separation based on stimulation time and modality. Each dot represents and individual sample in this experiment: 7DIV Control *n* = 3; 7DIV Bic15 min *n* = 3; 7DIV Bic180 min *n* = 3; 7DIV KCl15 min *n* = 3; 7DIV KCl180 min *n* = 3; 21DIV Control *n* = 3; 21DIV Bic15 min *n* = 2; 21DIV Bic180 min *n* = 2; 21DIV KCl15 min *n* = 3; and 21DIV KCl180 min *n* = 3.

To understand how these differences are translated into activity-driven transcription, we evaluated gene expression after 15 min and 180 min stimulation with KCl and Bic followed by RNA sequencing (Figure 2A). At 7DIV, KCl stimulation induced 443 DEG compared to unstimulated controls, while synaptic induction of neuronal activity using Bic only produced the induction of 39 DEG (Figure 2B, Supplementary Table S2). In 21DIV neurons, both types of stimulation induced high numbers of DEG: 1245 with KCl and 1810 after synaptic stimulation with Bic (Figure 2B, Supplementary Table S2). By comparing the DEG shared between stimulation conditions, we observed a large proportion of the genes exclusively induced in each condition, indicating specificity in the transcriptional response (Figure 2C, Supplementary Table S2). In addition, the proportion of shared DEG is higher between KCl/Bic in 21DIV neurons than DEG shared upon each stimulation modality at different stages (Figure 2C, Supplementary Table S2). PCA also separated 7DIV and 21DIV samples depending on stimulation modality and time (Figure 2D).

**Figure 3:**
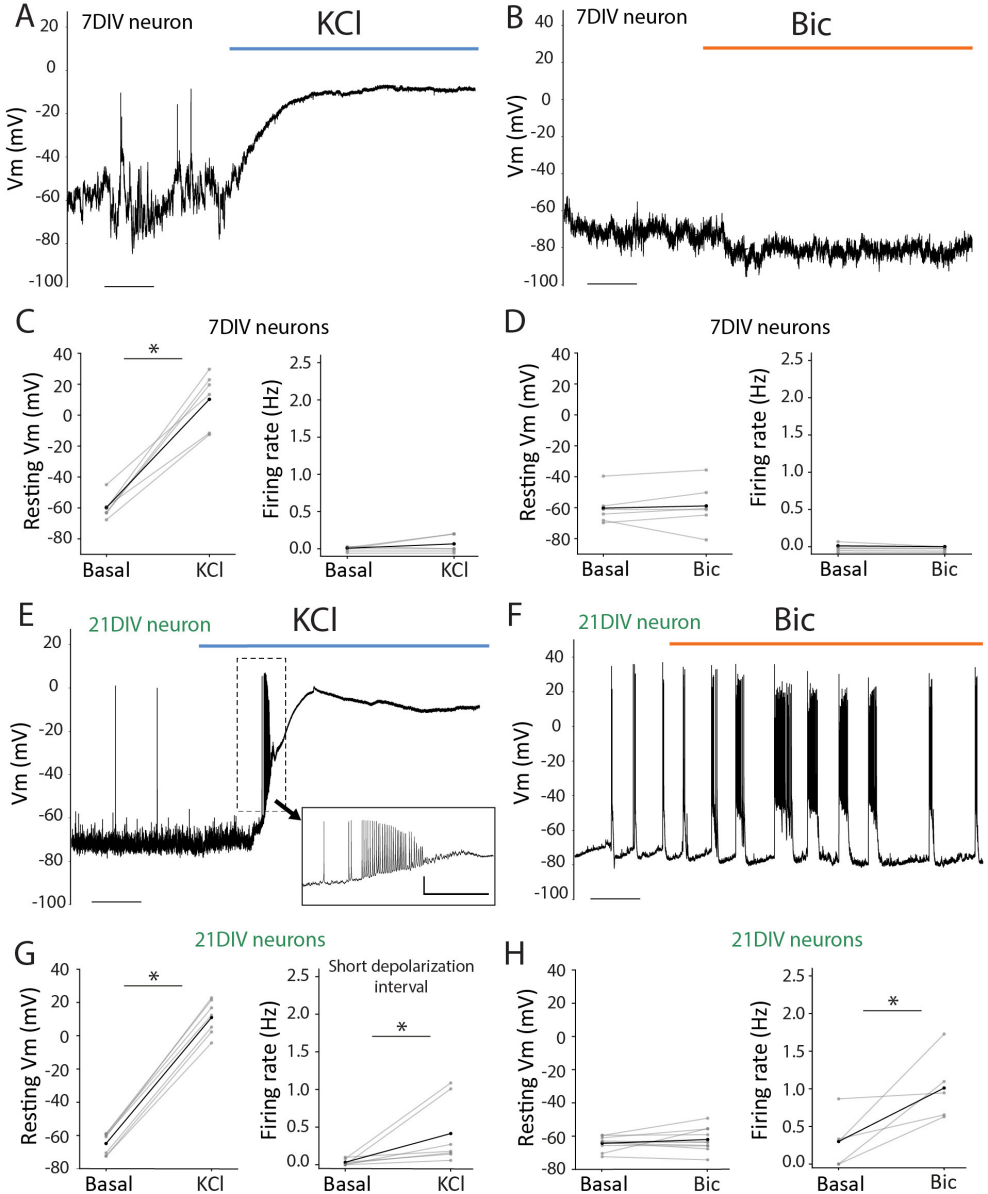
Electrophysiological response to KCl or Bic induction in 7DIV and 21DIV. **(A)** Representative whole-cell patch-clamp recordings at 7DIV during KCl administration. Scale: *t* = 50 s. **(B)** Representative whole-cell patch-clamp recordings at 7DIV during Bic administration. Scale: *t* = 25 s. **(C)** Left: voltage membrane (Vm) measurements of 7DIV neurons prior to and after KCl administration. Paired student's *t*-test: t-statistic= −9.355, *p* = 0.0002; *n* = 6. Rigth: firing rate (Hz) 7DIV neurons prior to and after KCl administration. Paired student's *t*-test: t-statistic= −1.371, *p* = 0.2288; *n* = 6. **(D)** Left: voltage membrane (Vm) measurements of 7DIV neurons prior to and after Bic administration. Paired student's *t*-test: t-statistic= −0.469, *p* = 0.6589; *n* = 6. Rigth: firing rate (Hz) 7DIV neurons prior to and after Bic administration. t-statistic = 1.088, *p* = 0.3263; *n* = 6. **(E)** Representative whole-cell patch-clamp recordings at 21DIV during KCl administration. Inset magnification illustrates a representative train of action potentials observed in these recordings at the beginning of depolarization. Scales: *t* = 50 s (main); and *t* = 5 s; *v* = 20 mV (inset). **(F)** Representative whole-cell patch-clamp recordings at 21DIV during BIC administration. Scale: *t* = 25 s. **(G)** Left: voltage membrane (Vm) measurements of 21DIV neurons prior to and after KCl administration. Paired student's *t*-test: t-statistic: −16.498, *p* = 1.30e-9; *n* = 7. Rigth: firing rate (Hz) 21DIV neurons comparison between pre-stimuli and a short window during depolarization. Paired Student's *t*-test: t-statistic= −2.299498, *p* = 0.04e-9; *n* = 7. **(H)** Left: voltage membrane (Vm) measurements of 21DIV neurons prior to and after Bic administration. Paired Student's *t*-test: t-statistic: −1.207, *p* = 0.258; *n* = 10. Rigth: firing rate (Hz) 21DIV neurons prior to and after Bic administration. Paired Student's *t*-test: t-statistic= −2.877, *p* = 0.045; *n* = 5. ^*^means that the differences between the 2 groups are significant with a *p* value < 0.05.

The differences in activity-driven response might not be only due to the substantial molecular differences in the basal starting point (Figure 1) but also due to the distinct ability of the neurons to physiologically respond to each stimulation at these two maturational time points. To investigate this, we performed patch-clamp recordings on cultured neurons and evaluated their electrical responses during the administration of KCl or Bic. At 7DIV, KCl application induced no changes in the firing rate but resulted in a massive and sustained depolarization (Figures 3A, C). In contrast, Bic application did not alter neuronal activity (Figures 3B, D), consistent with our previous observations of minimal changes in activity-dependent gene expression at this stage. The electrophysiological profile at 21DIV differed markedly from that at 7DIV. KCl application initially triggered a burst of action potentials (Figure 3E), causing a transient increase in firing rate (Figure 3G) and, in all cases, also led to an irreversible shift in the membrane potential (Vm) toward a depolarized state (Figure 3G). In contrast, Bic stimulation increased the firing rate in a subset of neurons (Figures 3F, H) without inducing significant changes in Vm (Figure 3H). These findings demonstrate that neuronal firing elicits distinct responses depending on the maturational stage. Altogether, these results highlight that neuronal maturation is associated with significant alterations in gene expression, particularly in genes involved in synaptic organization and function, which are likely linked to the observed changes in neuronal activity.

### Widely used stimulation protocols elicit specific and non-equivalent transcriptional responses

**Figure 4:**
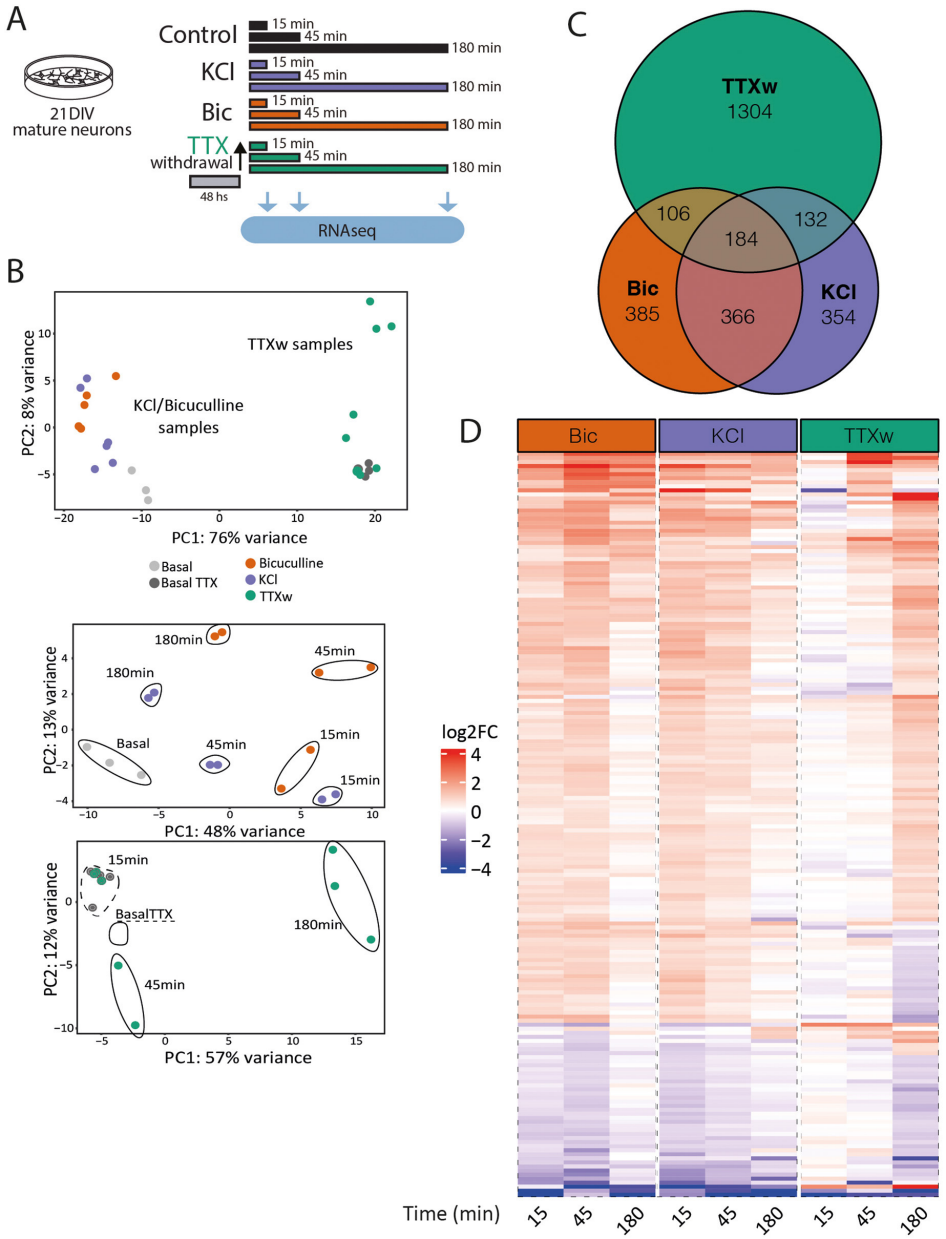
Gene expression programs induced by different neuronal activation modalities in mature neurons. **(A)** Experimental design: primary cortical neurons at 21DIV were stimulated with KCl, Bic, or TTXw for 15 min, 45 min, or 180 min, and RNA was extracted and sequenced to assess differential gene expression. **(B)** Gene expression PCA. Top: samples (including non-stimulated controls) cluster into two groups: KCl and Bic vs. TTXw samples. Bottom: PCA of these two groups shows a separation based on stimulation duration. Each dot represents and individual sample in this experiment: Basal *n* = 3; Bic15 min *n* = 2; Bic45 min *n* = 2; Bic180 min *n* = 2; KCl15 min *n* = 2; KCl45min *n* = 2; KCl180 min *n* = 2; BasalTTX *n* = 4; TTXw15 min *n* = 3; TTXw45min *n* = 2; and TTXw180 min *n* = 3. **(C)** Venn diagram of DEGs among stimulation modalities. **(D)** Heat map: fold induction levels (in log2) of the 184 shared DEG among stimulation modalities. Genes are grouped and ordered according to similar behaviors and dynamics.

Building on the observed differences, we aimed to systematically analyze how the transcriptional profile and dynamics behave in 21DIV neurons in response to acute stimulation by different stimuli. To investigate the temporal dynamics of gene expression, 21DIV neuronal cultures were exposed to KCl, Bic, or Tetrodotoxin removal (TTXw), and RNA sequencing was performed at three time points: 15, 45, and 180 min (Figure 4A). Although these methods are commonly used to activate neurons, their mechanisms of inducing activity differ significantly. Compared to KCl and Bic stimulation (Figures 3E–H), TTXw at 21DIV neurons also showed a specific activation patterns when removing the TTX after 48 h (Supplementary Figure 1A) with a significant increase in the firing rate (Supplementary Figure 1B). PCA of these transcriptomic data showed a clear separation of samples exposed to TTXw from the KCl- and BIC-treated cells (Figure 4B, upper panel). This result suggests that prior exposure to TTX for 48 h induces a silencing activity that leads to a distinct transcriptional state compared to KCl or BIC, indicating that different activation modalities are not equivalent. When we performed a separate PCA for KCl and Bic (grouped) and TTXw, we observed differences in the temporal dynamics of transcription across the different stimulation conditions (Figure 4B, lower panels). A total of 2,831 differential transcripts were found to be differentially expressed with respect to their basal condition: 1,726 DEG upon TTXw, 1,041 were induced by Bic, and 1,036 after KCl membrane depolarization (Figure 4C). Some DEG are shared among each group, with a greater proportion of genes shared between Bic and KCl when compared to TTXw (Figure 4C). A heatmap of the 184 DEGs common to all three conditions further illustrates the distinct gene expression profiles and dynamics (Figure 4D, Supplementary Table S3). Collectively, these data showed that differences are related to which genes are differentially expressed in each situation and the temporal dynamics of gene expression induced by each neuronal activity modality.

**Figure 5:**
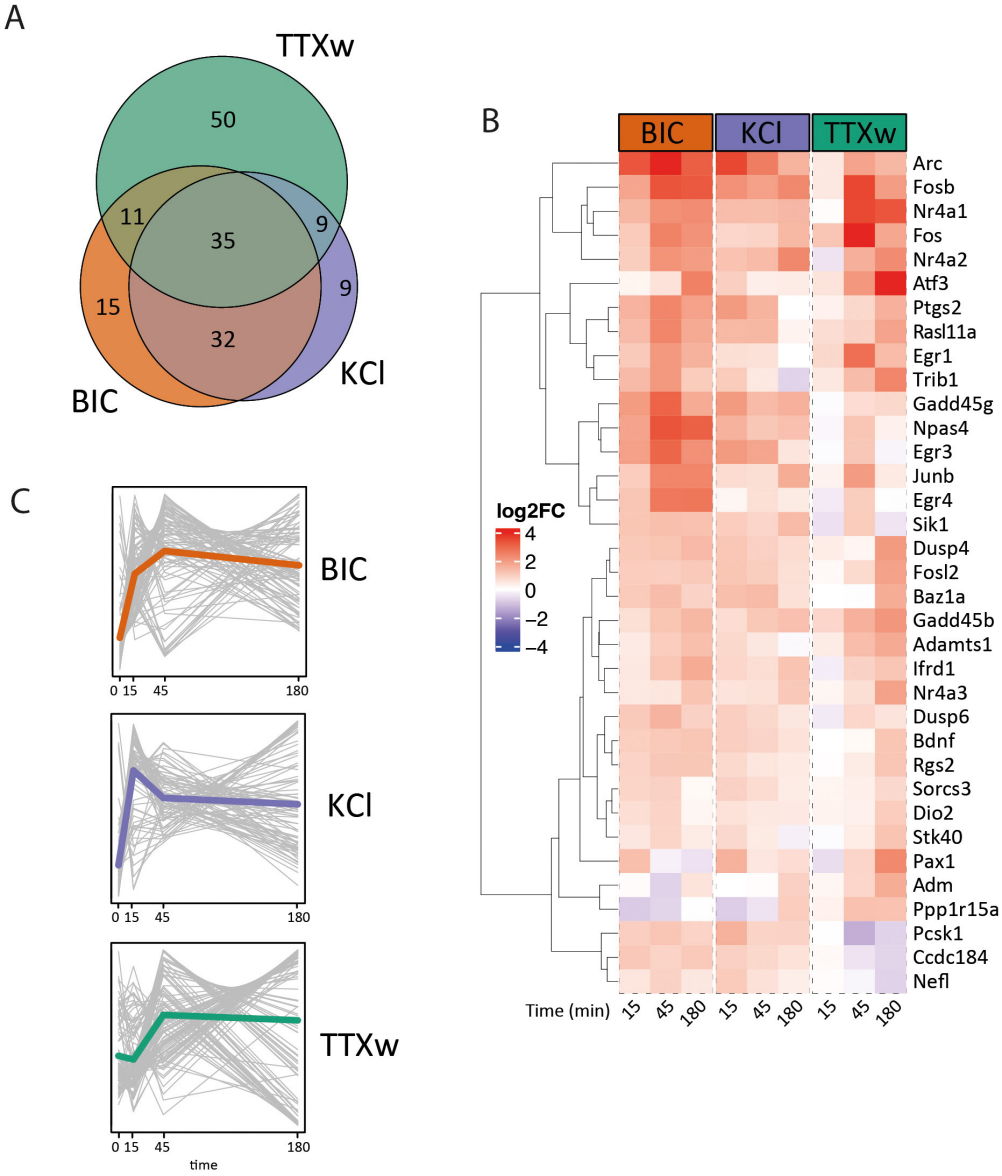
Immediate early gene expression dynamics. **(A)** Venn diagram: overlapping IEGs differentially expressed among stimulation modalities. **(B)** Heat map: fold induction levels (in log2) of 35 IEG differentially expressed upon stimulation modalities. Genes are grouped and ordered according to similar behaviors and dynamics. **(C)** Average IEG expression dynamics. Each gray line represents a gene, and the colored line indicates the genes' average trajectory corresponding to each dynamic.

To analyze immediate early genes' expression dynamics, we selected 300 previously described IEGS by Kim et al. (2010) and Tyssowski et al. (2018) and performed a cross-comparison of the selected candidates among the different treatments. Out of these 300 IEGs, we observed a total of 161 DEG in response to some of the stimulation modalities: 35 of these genes were induced upon every stimulus, 74 genes were induced exclusively by one stimulus, and 54 genes had their expression levels altered by two of the protocols −32 by Bic and KCl, 11 by Bic and TTXw, and 9 by KCl and TTXw- (Figures 5A, B). Nine IEGs changed only upon KCl stimulation, 15 under BIC and 50 genes were exclusively modified by TTXw. The set of 35 shared IEGs whose expression levels changed in all treatments includes many of the most conspicuous and best-studied neuronal relevance IEGs such as the members of the Fos family (Fosb, Fos, Junb, Fosl2), Npas4, Arc, Bdnf, Nr4a2, Nr4a3, Egr3, Egr4, Gadd45b, and Gadd45g, among others (Figure 5B). Interestingly, we observed distinct expression dynamics for these shared genes depending on the stimulation modality (Figure 5C). This collection of IEGs may represent a biologically relevant yet nonspecific gene transcriptional core that consistently responds to various activity-related stimuli, forming a basic ensemble of activity-responsive genes in neurons.

### Analysis of gene expression dynamics analysis and GO characterization

**Figure 6:**
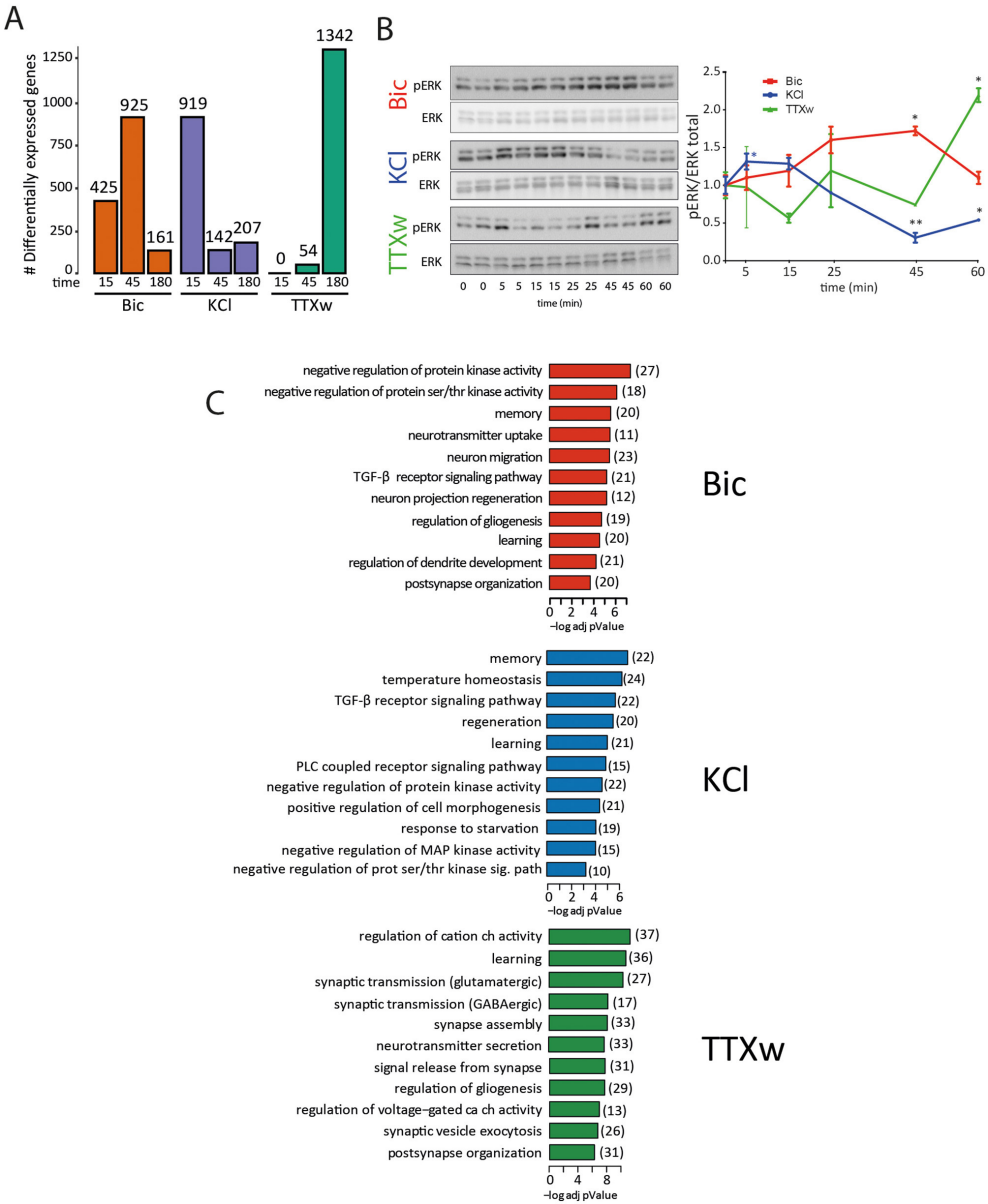
Gene expression dynamics analysis and ERK phosphorylation response to different stimuli. **(A)** Bar graph showing the number of DEG at each time point. **(B)** ERK phosphorylation kinetics upon stimulation treated with BIC, KCl, and TTXw for different times. Representative western blot for phosphorylated ERK (pERK) and total ERK. The dynamics of pERK relativized to total ERK are shown in the graph. Statistical comparison was made internally in each treatment, comparing with respect to the initial state at time zero. *p < 0.05, **p < 0.01, one-way ANOVA followed by multiple comparisons via Dunnett's test, *n* = 2. **(C)** Gene ontology enrichment analysis. Biological processes enriched in Bic, KCl, and TTXw experimental groups are shown. In each stimulation modality, genes from the time point with more DEG were used for this comparison. The number of DEGs in each biological process is reported in brackets.

We performed an additional analysis comparing DEG at each time point vs. the basal gene expression values to better understand global gene expression dynamics among stimulation protocols. Notably, differences in the number and dynamics of DEG were observed among treatments: Bic generated a peak in the number of DEG after 45 min; KCl depolarization induced a rapid gene induction of 925 DEG at 15 min; and, upon TTXw, changes in the number of DEG were strongly observed only after 180 min from drug withdrawal (Figure 6A, Supplementary Table S4). These results indicate that each stimulation protocol induces distinct temporal dynamics of gene expression.

It has been previously shown that the ERK1/2-MAPK pathway is a key signaling hub orchestrating activity-induced gene-expression programs (Ha and Redmond, 2008; Ohe et al., 2022; Tyssowski et al., 2018). Thus, ERK1/2 phosphorylation can be used as a molecular proxy to assess the link between the induction methods and its downstream gene expression programs. Western blot analysis revealed ERK1/2 phosphorylation dynamics correspond with the peaks observed in gene expression upon each stimulation (Figure 6B). Bic stimulation induced a significant increase in ERK1/2 phosphorylation at 45 min (one-way ANOVA followed by multiple comparisons via Dunnet's test: pERK1/2 Bic 45 min vs. pERK1/2 Basal 0 min, *p* = 0.0455), KCl stimulation generated an initial increase in ERK1/2 phosphorylation at 5 min, followed by a subsequent decrease (one-way ANOVA followed by multiple comparisons via Dunnet's test: pERK1/2 KCl 5 min vs. pERK1/2 Basal 0 min, *p* = 0.0494; pERK1/2 KCl 45 min vs. pERK1/2 Basal 0 min, *p* = 0.0016; pERK1/2 KCl 60 min vs. pERK1/2 Basal 0 min, *p* = 0.0131), and TTXw resulted in a significant increase at 60 min post-stimulation (one-way ANOVA followed by multiple comparisons via Dunnet's test: pERK1/2 TTXw 60 min vs. pERK1/2 Basal 0 min, *p* = 0.0482). Total ERK1/2 protein levels were largely unaffected. These findings indicate that each mode of neuronal activation triggers an ERK1/2 distinct activation pattern temporally aligned with the respective gene expression programs, suggesting that ERK1/2 phosphorylation might directly influence the timing of downstream transcriptional responses.

Time-course analysis also allows us to differentiate between up-regulated and downregulated genes. Notably, the proportion of up-regulated to downregulated genes varies across time points (Supplementary Figure 2A, Supplementary Table S4). Interestingly, the time points with the highest number of DEG for each stimulus coincide with both the peak of up-regulated genes and the highest proportion of downregulated DEG. This result suggests a coordinated regulation of gene expression, where both upregulation and downregulation occur in a stimulus- and time-dependent manner.

To uncover the functional significance of the DEGs, we conducted a Gene Ontology (GO) functional enrichment analysis. Examining the enriched GO biological processes for each experimental condition, we found a direct relationship between the number of DEGs and the corresponding enriched GO terms (Figure 6C, Supplementary Table S5). Notably, following TTXw treatment, the enriched GO terms included a higher proportion of downregulated genes, as indicated by the z-score (Supplementary Figure 2B). While general neuronal-related processes such as “learning” and “memory” were enriched across all treatments, specific neuronal and cellular GO terms were uniquely enriched depending on the stimulation modality (Figure 6C, Supplementary Table S5). Bic and TTXw treatments significantly enriched biological processes related to neuronal projections, dendritic projections, and synaptic organization. In contrast, these terms were not enriched following KCl treatment, which aligns with its mechanism of inducing a large influx of calcium without continuous synaptic activation. Overall, these findings further support the existence of stimulus-specific gene expression programs in neurons.

### Individual gene behavior and gene dynamic clustering

**Figure 7:**
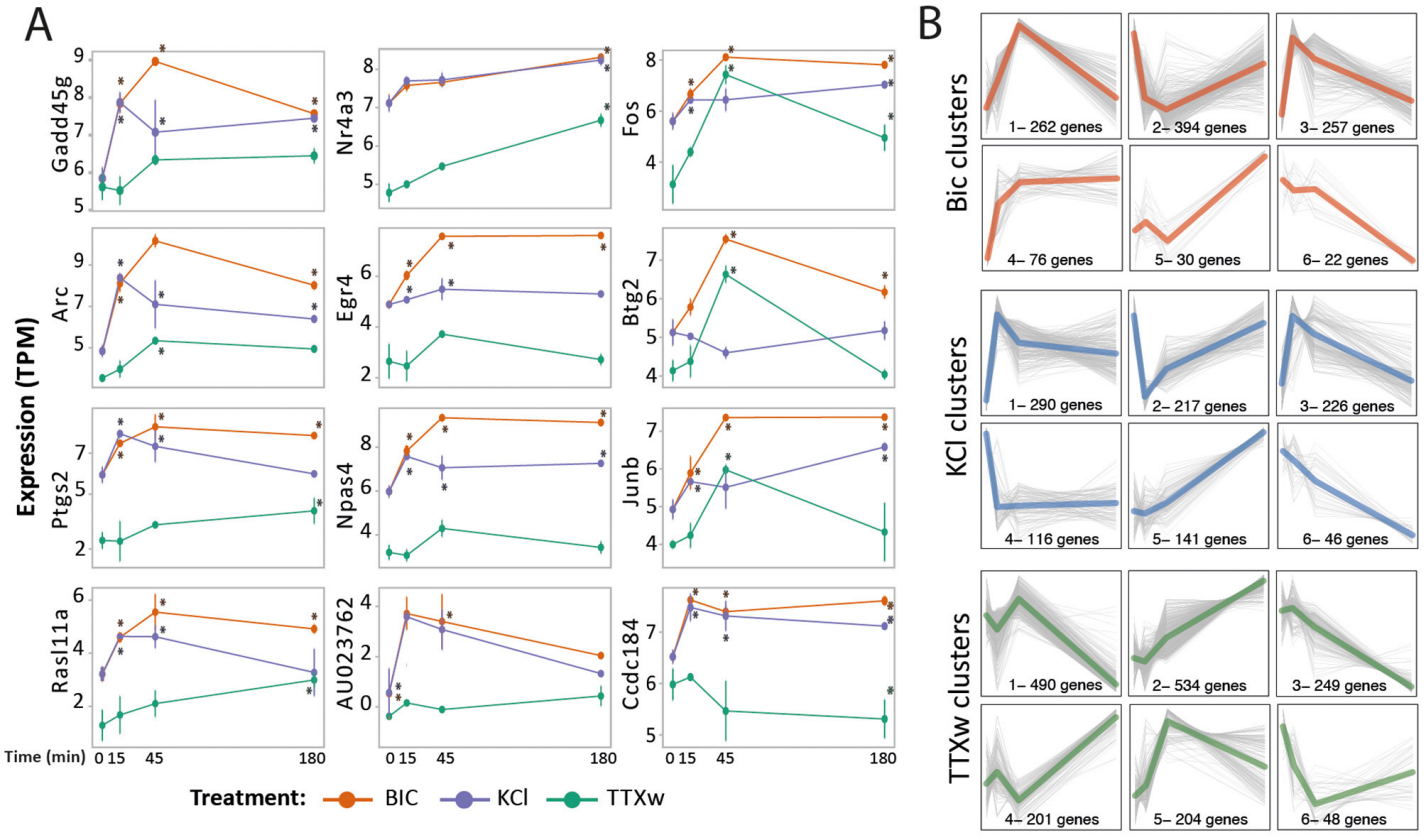
Individual gene behavior and gene dynamic clustering. **(A)** Gene expression dynamics: selected genes' expression levels are shown (TPM). Three gene groups with specific behaviors are presented: in the first column, transcripts peaking at the most prioritized time point for each stimulus (see Figure 5A); in the second column, genes responding similarly across the stimulation modalities; and in the third column, genes behaving differently upon each stimulus. All presented genes exceed the statistical significance filters in their global dynamics. Asterisks indicate significance vs. statistical measure of time-to-time comparison. **(B)** Gene dynamics clustering of DEG upon each stimulation modality. The dynamic-tree cut method was used to infer clusters from the obtained hierarchical dendrogram and those clusters whose mean profiles had a distance less than 0.3 were merged. Each gray line represents a gene, and the colored line is the genes' average trajectory corresponding to said dynamics. The number of genes that each of the six clusters of each treatment is indicated.

To gain deeper insight into individual gene temporal dynamics, we analyzed expression patterns at the single-gene level and identified three distinct groups with unique temporal profiles. The first group, including genes like Gadd45g, Arc, Ptgs2, and Rasl11a, exhibited expression patterns that aligned with the specific peaks for each stimulation modality (KCl at 15 m, BIC at 45 m, TTXw at 180 m shown in Figures 6A, B; Figure 7A, first column). The second group, comprising genes such as Nr4a3, Egr4, Npas4, and AU023762, showed a similar response pattern to the three different activation treatments (Figure 7A, second column). In contrast, genes of a third group, including Fos, Btg2, Junb, and Ccdc184, displayed distinct responses to each stimulus (Figure 7A, third column). These results underscore the complexity and diversity of temporal gene expression dynamics in response to different neuronal activation modalities.

Using the dynamic tree-cut method, we performed gene clustering of DEGs based on their expression dynamic and identified six different gene expression dynamics associated with specific neuronal activity patterns (Figure 7B, Supplementary Table S6). Most clusters exhibited a transient increase in gene expression with a peak at 15 or 45 min, followed by a return to initial levels (e.g., BIC_1 and BIC_3; KCl_1 and KCl_3; TTXw_1 and TTXw_5). In contrast, some clusters showed sustained changes in gene expression up to 180 min (e.g., BIC_4 and BIC_6, KCl_5 and KCl_6, TTXw_2 and TTXw_3). A third group of clusters displayed a marked change in gene expression direction at the intermediate time points (e.g., BIC_5, TTXw_1 and TTXw_4). Notably, clusters BIC_1 (peak at 45 min), KCl_1 (peak at 15 min), and TTX_2 (constant increase up to 180 min) contained the highest proportion of genes among each treatment, consistent with the distribution of DEG observed at each time point and treatment (Figure 6A). To evaluate gene responses and identify clusters of similar genes, we compare cluster composition along each stimulation condition (Supplementary Figure 3). BIC_2/KCl_2 pair shares the most genes, with their dynamics closely aligned (Figure 7B). Other pairs, such as BIC_3/KCl_1, BIC_3/KCl_3, BIC_1/KCl_1, and BIC_1/KCl_3, share 60–100 genes and display similar dynamics, highlighting substantial overlap between BIC and KCl treatments. TTXw clusters also overlap significantly with BIC and KCl, particularly TTXw_2, which shares genes with BIC_1, BIC_3, KCl_1, and KCl_3. However, TTXw_2 exhibits distinct dynamics, with sustained gene expression increases up to 180 min (Supplementary Figure 3A). Among the 184 DEGs shared across all activation paradigms, the most gene-rich combinations included BIC_1/KCl_1/TTXw_2, BIC_3/KCl_1/TTXw_2, BIC_1/KCl_3/TTXw_2, and BIC_3/KCl_3/TTXw_2 (Supplementary Figure 3B). Genes with reduced expression were prominent in BIC_2/KCl_2/TTXw_3 and BIC_2/KCl_2/TTXw_1.Add (Supplementary Figure 2B).

Our gene clustering analysis revealed distinct gene expression dynamics corresponding to specific neuronal activity patterns, providing valuable insights into the diversity of transcriptional responses to various modes of neuronal activation. These findings offer significant implications for understanding the regulatory mechanisms underlying neuronal stimuli responses.

## Discussion

Previous research defined the timeline of synaptogenesis in primary neuronal cultures, showing that synapse formation begins around DIV7-12 (depending on culture conditions), reaching maturation 6–10 days later (Banker, 2018; Benson and Cohen, 1996; Harris et al., 1992; Kaech and Banker, 2006; Li and Sheng, 2003; Papa et al., 1995). In fact, not only the structure but also the functional matching between the pre- and postsynaptic compartments increases with neuronal maturation (Kay et al., 2011). The morphological and electrophysiological measurements of our cultured neurons indicate that they follow the same timeline (Figures 1, 3). In particular, the absence of an evoked electrophysiological response to Bic-induced synaptic stimulation in DIV7 neurons (Figure 3B), in contrast to the robust response in DIV21 neurons (Figure 3F), suggests that functional synapses are largely absent at this developmental stage in our culture conditions. Accordingly, a comparative analysis of DIV7 vs. DIV21 neurons revealed substantial transcriptional differences in basal gene expression, with 3.885 genes differentially expressed between the two populations (Figure 1). Even though DIV7 neurons remain largely unresponsive to (Bic-induced) synaptic transmission, they do respond to KCl, although with a smaller gene expression response (443 DEG at DIV7 vs. 1,245 DEG at DIV21) (Figure 2). Interestingly, these differences are not only quantitative but also qualitative, as only 115 common genes are differentially expressed at both developmental stages (Figure 2). KCl treatment bypasses synaptic transmission, directly triggering action potentials in each neuron. Thus, the reduced sensitivity to KCl in DIV7 neurons indicates that signaling cascades and transcriptional machinery necessary to translate activity into gene expression programs are still immature and not fully functional at this early stage. These findings hold relevance for a vast research landscape of studies addressing activity-related phenomena where experiments involving primary neurons treated with KCl at DIV7-12 are abundant. Therefore, our results underscore the importance of considering neuronal developmental stage when interpreting transcriptional responses to activity stimulation and suggest that standardizing the use of more mature neuron cultures—such as 21DIV—may improve physiological relevance in future research.

It is also important to highlight that KCl treatment protocols remain unstandardized in the field. As thoroughly reviewed by Rienecker et al. (2020), key experimental variables—including KCl concentration, duration of exposure, composition of the treatment solution, media replacement procedures, and prior neuronal silencing—can substantially influence intracellular signaling dynamics and downstream transcriptional outcomes (Rienecker et al., 2020; Wheeler et al., 2008). While the use of KCl, Bicuculline, and other stimulation methods, has been extensively described, it is important to emphasize that these experiments are conducted in *in vitro* neuronal networks. For instance, the basal activity state of recurrent networks in DIV21 neuron cultures likely differs from that of native circuits, potentially affecting both the baseline gene transcriptional state and the magnitude of activity-induced gene expression changes. These inherent limitations of the model naturally constrain data interpretation, and any direct comparisons or extrapolations to *in vivo* systems should be approached with caution. In this context, the pharmacological activation of neurons in culture, while effective, reflects conditions that are likely extreme or non-physiological relative to the complexity of *in vivo* neuronal activity. Nonetheless, *in vitro* approaches have been successful as a hypotheses generator and in identifying key immediate early genes (IEGs) (Ataman et al., 2016; Bading et al., 1993; Boulting et al., 2021; Flavell and Greenberg, 2008; Kim et al., 2010; Lin et al., 2008; Malik et al., 2014; Mardinly et al., 2016; Sharma et al., 2019; Spiegel et al., 2014; Stroud et al., 2017; Yun et al., 2013) whose functional relevance has subsequently been validated *in vivo*. Importantly, the identification of activity-regulated mRNAs directly from brain tissue has helped overcome some limitations of *in vitro* systems and significantly advanced our understanding of IEG induction *in vivo*. This has been achieved in model organisms such as flies—using temperature or light-controlled neuronal activation (Chen et al., 2016)—, and rodents, through protocols including intense light exposure after prolonged dark housing (Hrvatin et al., 2018; Tyssowski et al., 2018; Xu et al., 2021), the induction of epileptic-type electrical activity (Fernandez-Albert et al., 2019; Lacar et al., 2016), or stimuli associated with the administration of abuse drugs (Mukherjee et al., 2018; Muniz et al., 2017). In fact, these studies described a broad range of transcriptional responses and IEG induction. A comparative analysis of that data reveals the presence of a core set of genes consistently emerging across various stimuli, including Egr1, Egr4, Egr2, Npas4, Nr4a1, Dusp1, Fos, Arc, Jun, Fosb, Btg2, Atf3, Junb, Gadd45g, Nr4a2, Ier5, and Irs2. Notably, we identified a core set of 35 IEGs responsive to all three activation stimuli tested, encompassing many of these responsibly conserved genes (Figure 5). This suggests the existence of a core transcriptional program governing the genetic response to neuronal activation, irrespective of the specific intracellular mechanisms involved. However, the activation of this core gene set does have a stimulus-dependent temporal dynamics, revealing a complex interplay between shared and specific signaling pathways. This implies that the temporal patterns of gene induction serve as a molecular fingerprint, encoding information about the underlying mechanisms driving neuronal activation.

Furthermore, neuronal activity to gene expression mechanisms can be conceptualized as a translation of digital signals (e.g., pulses/discrete patterns) into analog intracellular signals. This process has been previously described in non-neuronal cells such as NIH 3T3 and PC12 cell lines showing that pulsatile stimulus over ligand or light-induced receptor activation can not only initiate distinct signaling cascades but also generate different activation dynamics of the same kinase pathway, such as ERK which subsequently controls IEG expression in those cell systems (Ravindran et al., 2022, 2020; Toettcher et al., 2013; Wilson et al., 2017). In fact, ERK is known to act as a central regulator of IEG expression in neurons (Tyssowski et al., 2018). Remarkably, ERK activation dynamics closely paralleled the temporality of activity-dependent gene expression observed across KCl, Bic, and TTXw stimulation (Figure 5B), indicating that transcriptional responses are, at least in part, governed by the precise timing of early intracellular signaling events.

For years, KCl, Bic, and TTXw have been used as methods to induce neuronal activity under the consideration that they produce equivalent effects. Recently, emerging evidence has also shown the divergent impacts of these stimuli on specific cellular phenomena. For instance, Bic and TTXw lead to distinct effects on nucleus-synapse transport (Ch'ng et al., 2012), while Bic, KCl, and TTXw induce different Arc transcriptional bursts (Das et al., 2018). However, despite their widespread use, no comprehensive comparison of these activation modalities has been conducted to elucidate their specific roles in activation-regulated transcription. Our analysis revealed marked disparities in the gene expression programs triggered by the different stimuli. This underscores the importance of considering Bic, KCl, and TTXw as non-equivalent, unique activation modalities to avoid misinterpretations and enhance reproducibility across different fields in neuroscience, studying diverse aspects of neuronal activation.

A more precise approach to dissecting the effects of activity patterns might be the use of electrodes providing direct electrical stimulation over neuronal cultures. Previous studies in dorsal root ganglion (DRG) neurons have shown that diverse bursting electrical stimulation patterns can induce specific gene expression profiles (Iacobas et al., 2019; Lee et al., 2017; Sheng et al., 1993). However, the magnitude of expression changes observed is relatively modest, possibly due to the extended activation protocols used (up to 5 h). It is important to note that these studies were conducted in DRG neurons that develop in absence of synaptic contacts and remain silent in culture.

The difficulties in evaluating activity-dependent gene expression changes in response to short-duration controlled stimuli explain the predominance in this field of research of protocols that stimulate neuronal activity for longer durations like KCl, Bic, and TTXw with less control over the firing frequencies of neurons. Attempts to perform controlled-frequency stimulations were also performed using optogenetics in neuronal cultures. For example, few studies have demonstrated precise temporal control of IEG induction, such as Fos, by light in neurons expressing ChR2 (Schoenenberger et al., 2009). Furthermore, recent studies have demonstrated that *in vitro* neurons can reliably follow optogenetic stimulation frequencies up to 10 Hz for several minutes, but fidelity declines at higher frequencies (Yang et al., 2024). However, optogenetic stimulation using light also has limitations. Notably, blue light alone can induce activity-dependent gene expression in neuronal cultures, even in the absence of channelrhodopsins in neuronal cultures (Tyssowski and Gray, 2019). These effects appear to result from phototoxic interactions with molecules present in neuronal culture media (Duke et al., 2020). To overcome this limitation, future studies could utilize electrical stimulation at specific frequencies of single pulses or bursts over varying time points using multi-electrode array (MEA) plates, in combination with pharmacological blockers of excitatory and inhibitory neurotransmission (e.g., APV-NBQZ or kynurenic acid plus picrotoxin). Such an approach would allow for the isolation of cell-intrinsic activation mechanisms while minimizing the contribution of network-level activity.

The advent of single-cell RNA sequencing, especially with anticipated improvements in sequencing depth, will be instrumental both in cultures and *in vivo* setups. In neuronal cultures, it would help to understand the magnitude and variability in responses among individual neurons in culture. *In vivo* studies combining neuronal labeling techniques with single-cell RNA sequencing will be crucial to pinpoint gene expression uniquely in neurons that were activated or incorporated into a memory engram (FOS+ or ARC+) in response to more physiological changes, such as exposure to a novel environment (Lacar et al., 2016) or engagement in a fear conditioning paradigm (Cho et al., 2016).

We also observed a substantial number of genes that were downregulated following neuronal activation, a phenomenon that remains largely understudied. One possible explanation is that this downregulation facilitates the upregulation of other genes essential for activity-driven neuronal changes. Although no specific strong shared identity emerged among the downregulated genes, the negative chromatin regulator Hdac11 (Liu et al., 2020) showed decreased expression following KCl, Bicuculline, and TTXw. Similarly, genes linked to autophagy, proteasome and endoplasmic-reticulum-associated degradation such as Derl3, Prss36, Atg2b, Ulk1, Ulk2, and Stbd1, were also downregulated. As were genes involved with mRNA degradation pathways, including Ufl1 and Piwil2, the latter of which functions with piRNA to methylate and silence gene targets (Rajasethupathy et al., 2012). These findings suggest a potential reduction in mRNA and protein degradation processes in neurons during activation. While we did not identify upregulated genes associated with transcriptional repression machinery, uncovering the role of this widespread gene downregulation offers an opportunity to deepen our understanding of complex regulatory mechanisms triggered by neuronal activity.

In summary, our study reveals that neuronal development significantly impacts the transcriptional response to neuronal activity. Using KCl, Bic, and TTXw to induce distinct neuronal activation patterns, we found that each stimulus elicits unique gene expression profiles in mature neurons. These findings emphasize the crucial need to consider both neuronal developmental stage and activation modality when exploring activity-dependent gene regulation.

## Data Availability

The datasets presented in this study can be found in online repositories. The names of the repository/repositories and accession number(s) can be found in the article/Supplementary material.

## Supplementary material

Supplementary Figure S1 Electrophysiological response to TTX withdrawal in 21DIV. **(A)** Representative whole-cell patch-clamp recordings at 21DIV during TTX withdrawal after 48 h of TTX treatment. Scale: *t* = 1 min. **(B)** Firing rate (Hz) comparison between 21DIV neurons basal firing rate and TTX withdrawal protocol induced firing rate. Paired Student's *t*-test: t-statistic= −5.126, *p* = 8.9e10^−06^; Basal TTX *n* = 30; TTXw *n* = 8.

Supplementary Figure S2 Gene expression dynamics analysis responses to different stimuli. **(A)** Table presenting the number and percentage of DEG with significant increase or decrease in expression levels. **(B)** Diagram showing the characteristics of GO biological processes found enriched in each experimental condition. Each circle represents a significantly enriched GO group. The color of the circles indicates the statistic value of that functional enrichment, while the size of the circles represents how many genes belonging to that group were found. Z-score estimates whether DEG present in each group are positively (z score > 0) or negatively (z score < 0) regulated compared to the basal situation.

Supplementary Figure S3 Gene clustering analysis. **(A)** Distributions of the number of genes corresponding to each dynamic clusters and proportion of shared genes among clusters of different treatments. Darker squares correspond to pair of clusters that have a greater number of genes in common. The color of each row or column indicates the number of genes in that cluster at one end and the treatment to which they correspond at the other. **(B)** Cluster trios with top amount of the 184 shared DEG upon Bic, KCl, and TTXw.
