# Supplementary figures and images for: Neuronal activity-dependent gene expression is stimulus-specific and changes with neuronal maturation
Source: Front Mol Neurosci. 2025 Oct 9;18:1609772. doi: 10.3389/fnmol.2025.1609772 (PMC12547137; doi:10.3389/fnmol.2025.1609772)

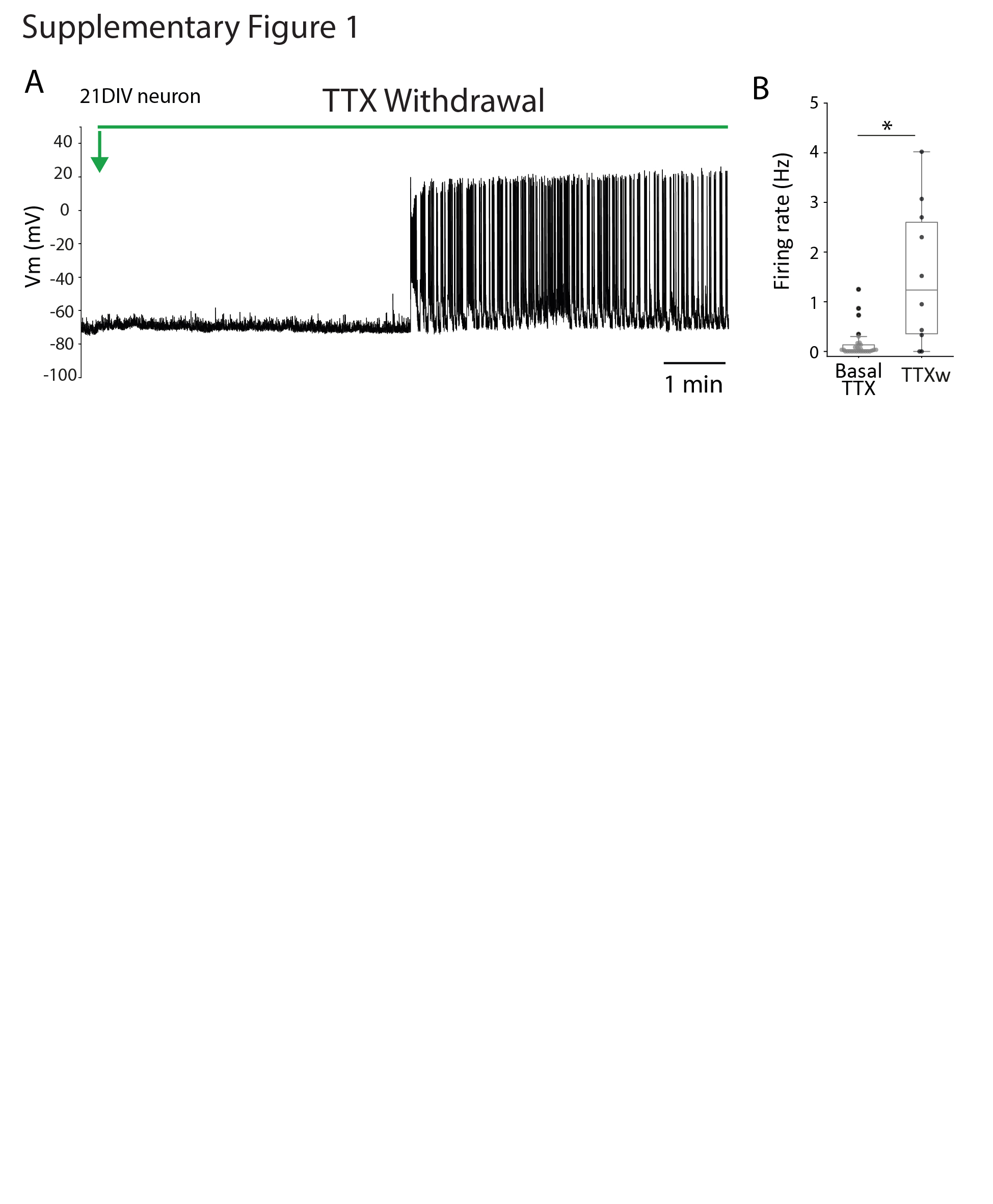

Supplement: Supplementary Figure S1 — Electrophysiological response to TTX withdrawal in 21DIV. (A) Representative whole-cell patch-clamp recordings at 21DIV during TTX withdrawal after 48 h of TTX treatment. Scale: t = 1 min. (B) Firing rate (Hz) comparison between 21DIV neurons basal firing rate and TTX withdrawal protocol induced firing rate. Paired Student's t-test: t-statistic= −5.126, p = 8.9e10−06; Basal TTX n = 30; TTXw n = 8. [file Image_1.jpg]

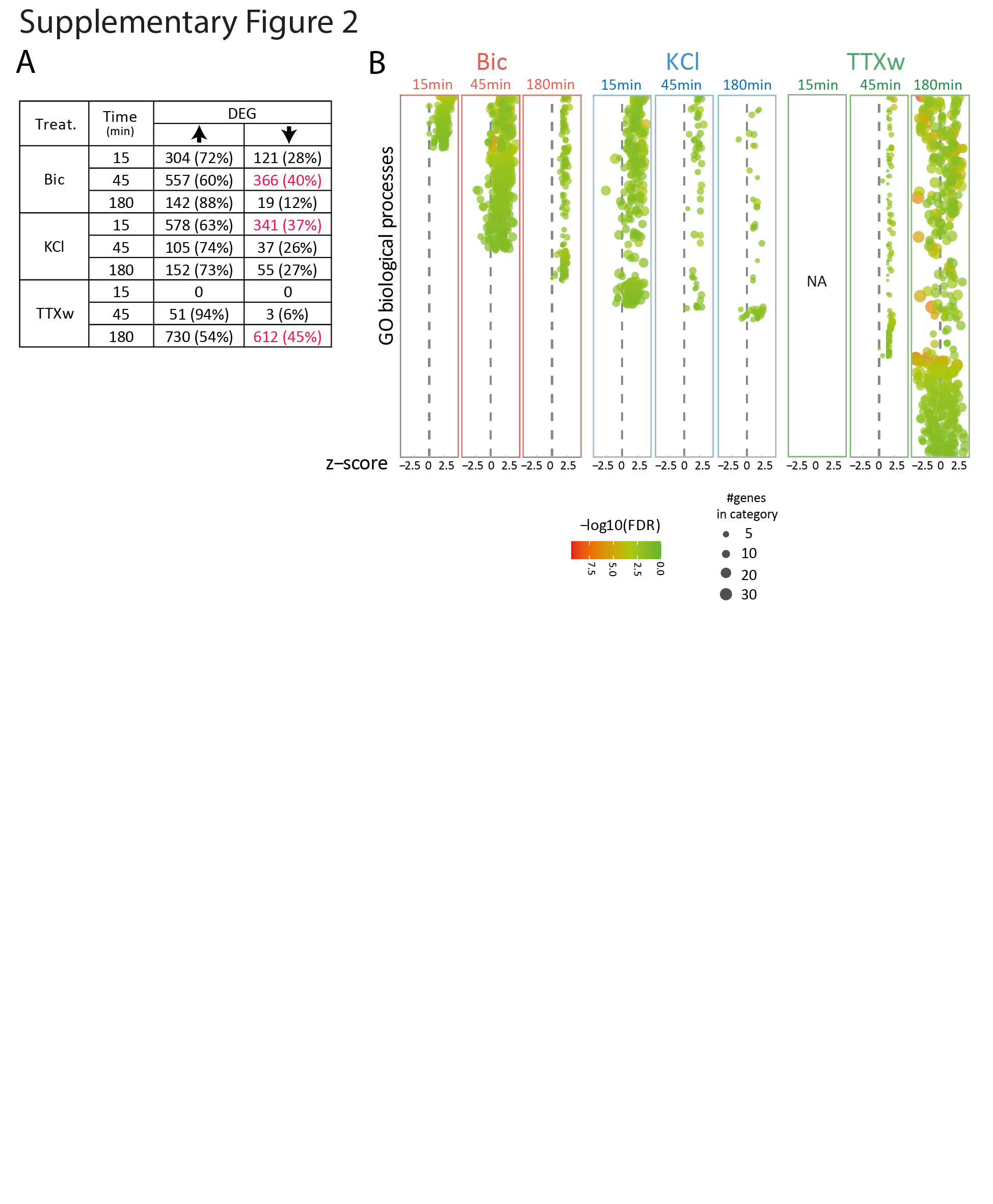

Supplement: Supplementary Figure S2 — Gene expression dynamics analysis responses to different stimuli. (A) Table presenting the number and percentage of DEG with significant increase or decrease in expression levels. (B) Diagram showing the characteristics of GO biological processes found enriched in each experimental condition. Each circle represents a significantly enriched GO group. The color of the circles indicates the statistic value of that functional enrichment, while the size of the circles represents how many genes belonging to that group were found. Z-score estimates whether DEG present in each group are positively (z score > 0) or negatively (z score < 0) regulated compared to the basal situation. [file Image_2.jpg]

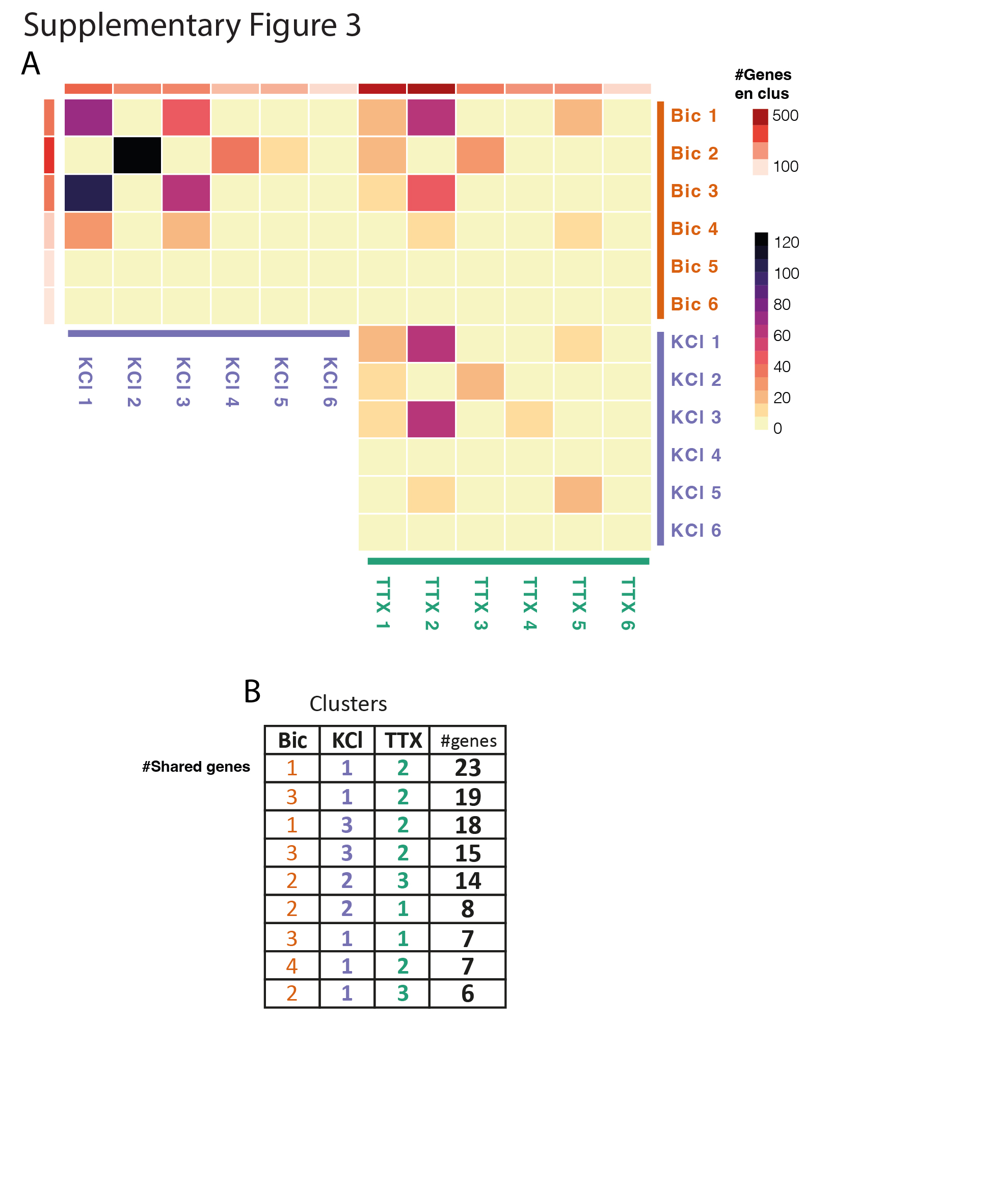

Supplement: Supplementary Figure S3 — Gene clustering analysis. (A) Distributions of the number of genes corresponding to each dynamic clusters and proportion of shared genes among clusters of different treatments. Darker squares correspond to pair of clusters that have a greater number of genes in common. The color of each row or column indicates the number of genes in that cluster at one end and the treatment to which they correspond at the other. (B) Cluster trios with top amount of the 184 shared DEG upon Bic, KCl, and TTXw. [file Image_3.jpg]
